# Human Induced Pluripotent Stem Cells Derived from a Cardiac Somatic Source: Insights for an In-Vitro Cardiomyocyte Platform

**DOI:** 10.3390/ijms21020507

**Published:** 2020-01-13

**Authors:** Alessandra Maria Lodrini, Lucio Barile, Marcella Rocchetti, Claudia Altomare

**Affiliations:** 1 Department of Biotechnology and Biosciences, Università degli Studi di Milano-Bicocca, Milano 20126, Italy; a.lodrini@campus.unimib.it (A.M.L.); marcella.rocchetti@unimib.it (M.R.); 2 Fondazione Cardiocentro Ticino, Lugano 6900, Switzerland; lucio.barile@cardiocentro.org; 3 Faculty of Biomedical Sciences, Università della Svizzera Italiana, Lugano 6900, Switzerland

**Keywords:** hiPSC-CMs, epigenetic memory, maturation, cell modelling, drug testing

## Abstract

Reprogramming of adult somatic cells into induced pluripotent stem cells (iPSCs) has revolutionized the complex scientific field of disease modelling and personalized therapy. Cardiac differentiation of human iPSCs into cardiomyocytes (hiPSC-CMs) has been used in a wide range of healthy and disease models by deriving CMs from different somatic cells. Unfortunately, hiPSC-CMs have to be improved because existing protocols are not completely able to obtain mature CMs recapitulating physiological properties of human adult cardiac cells. Therefore, improvements and advances able to standardize differentiation conditions are needed. Lately, evidences of an epigenetic memory retained by the somatic cells used for deriving hiPSC-CMs has led to evaluation of different somatic sources in order to obtain more mature hiPSC-derived CMs.

## 1. Introduction

Human induced pluripotent stem cells (hiPSCs) have assumed a pivotal role in research since their discovery in 2007 [1]. The possibility to differentiate them into functional cardiomyocytes (hiPSC-CMs) awakened excitement for the potential use of those cells in repairing and regenerating damaged cardiac tissue [2,3]; however, even though hiPSC-CMs represent an autologous source that overcomes the immunological limitations and ethical concerns belonging to embryonic stem cells (ESCs), the risk of tumor formation and uncontrolled differentiation have restricted this kind of approach. The possibility to characterize specific phenotypes associated with patient-specific genotypes allows the use of hiPSC-derived cells for disease modelling and drug development with very promising results [4,5,6].

Several works in the past have reported that hiPSCs are similar to ESCs, but it was recently demonstrated that, because of their somatic origin, epigenetic memory can influence their differentiation and maturation processes [7]. Furthermore, quite a few studies have demonstrated that hiPSC-CMs are molecularly and functionally immature and resemble embryonic and neonatal CMs [8,9,10,11]. Differences in structural morphology, gene and protein expression, as well as calcium handling and ionic patterns, have been described using a time-course of hiPSC-CMs maturation in-vitro; electrical properties and physiology of derived CMs can dramatically change in a time-dependent way, thus leading to the crucial need to optimize time and culture conditions during differentiation [12].

The focus of this review is to raise the issue of the different limitations and strengths affecting hiPSC-CMs derived from different somatic sources by the same patient, with particular attention to the role of cell origin and the advantages of CMs derived from a cardiac source.

## 2. Reprogramming

The discovery by Takahashi and Yamanaka in 2006 demonstrated that a defined set of factors is able to directly reprogram a somatic cell to an ESC-like state [13]. Out of 24 candidate ESC-associated genes, just four (i.e., *Oct4*, *Sox2*, *Klf4*, and *c-Myc*) have been determined sufficient to convert fibroblasts to a pluripotent cell type, iPSCs. These four “Yamanaka factors” were first constitutively expressed using retroviral vectors in both mouse [13] and human [1] fibroblasts, inducing these terminally differentiated cells to express genes that are typical of ESCs.

The original iPSCs reprogramming strategy is still being used and remains mostly unaltered, but some advances have been made in the delivery of the four “Yamanaka factors” to improve efficiency. iPSCs have been successfully generated using both integrating and non-integrating methods, but the latter seems to have advantages regarding safety due to a reduced risk of genotoxicity and insertional mutagenesis [14]. Integrating methods include retroviral [13] and lentiviral delivery [15], while non-integrating methods include Sendai viruses [16,17], episomal plasmid transfer [18,19], co-MIP [20], piggyBac transposons [21], small molecules [22], miRNAs [23], and protein-mediated delivery [24].

Many cell types have been successfully reprogrammed to pluripotency, including mononuclear cells from blood [25], umbilical cord and placenta [26], urine-derived cells [27], hair keratinocytes [28] and cardiac progenitor cells [29].

**Figure 1:**
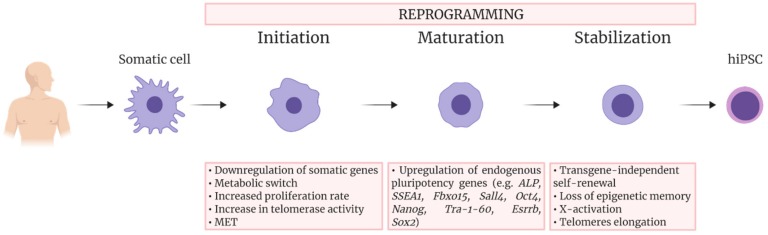
Schematic overview of the sequential events occurring during somatic cell reprogramming into human iPSCs. The process consists of three steps, Initiation, Maturation, and Stabilization. The main events occurring during each step are indicated.

The process to attain pluripotency has been described as consisting of three steps (Figure 1) [30,31]. The first one, called initiation, is characterized by the downregulation of signature somatic genes, a metabolic switch from oxidative phosphorylation to glycolysis, an increase in cell proliferation and reactivation of telomerase activity. This stage also requires changes in cell morphology, in particular a mesenchymal-to-epithelial transition (MET), which involves the acquisition of epithelial characteristics as cell polarity and expression of E-cadherin. These morphological changes are important since it is known that the cell shape itself is involved in epigenetic modifications regulating reprogramming [32].

The second phase of reprogramming, called maturation, involves the upregulation of endogenous pluripotency genes. These genes include the alkaline phosphatase, *SSEA1*, *Fbxo15*, *Sall4*, *Oct4*, *Nanog, Tra-1-60, Esrrb,* and finally *Sox2* [33]. The maturation step of reprogramming is likely the cause of the low efficiency of the reprogramming process and, indeed, a great number of cells in this phase undergo apoptosis or reversion [34].

Only 1% of the cells that initiate reprogramming make it to the third and final step, called stabilization; these are the cells that manage to repress transgene expression and activate endogenous pluripotency genes, becoming “stabilization-competent” [35]. Other changes occurring during the stabilization phase involve, for example, rearrangements in DNA methylation [33].

The core pluripotency gene cocktail is constituted by *Oct4*, *Sox2* and *Nanog*. These transcription factors form a circuitry for pluripotency which is autoregulatory, since all of them are able to regulate the expression of each other. *Oct4*, *Sox2,* and *Nanog* have the ability to activate genes necessary to maintain ESC-like pluripotency and to repress lineage-specific transcription factors, preventing the exit from the pluripotent state [36,37]. Other factors present in reprogramming cocktails, such as *c-Myc* or *Glis1*, are used to facilitate activation of this autoregulatory circuitry by stimulating gene expression and proliferation in general [38,39].

The original reprogramming strategy has been widely used, leading improvements in the cell reprogramming process. However, the translation of iPSC to a clinical setting is challenged by many obstacles, such as frequent incomplete reprogramming of the cells. Indeed, there are differences in the transcriptomes of iPSCs and ESCs and this may result from iPSCs either not activating pluripotency genes in the same way ESCs do, or not completely silencing somatic genes [40]. Moreover, de novo mutations may occur during the reprogramming process and the culture of generated iPSCs [41]. The lack of a rapid and precise test to evaluate the level of reprogramming in iPSCs aggravates this challenge.

To overcome these issues, an alternative approach that bypasses the pluripotent stage has been developed. This strategy, called transdifferentiation or direct reprogramming, allows for the reprogramming of one somatic cell type directly into another by delivery of single or multiple specific transcription factors of the desired lineage. Different studies have shown that, with this technique, fibroblasts can be directly converted to several other cell types including neurons [42], cardiomyocytes [43], endothelial cells [44], hepatocytes [45] and chondrocytes [46]. However, in these works transdifferentiation did not always translate to human cells as effectively as it does in murine cells [47]. Recently, it has been reported that fibroblasts from human donors can be efficiently converted to myoblasts by the overexpression of MYOD1 and MYCL [48,49]; the myotubes from this study seem a promising cell source for cell therapy when tested in-vitro, but have yet to be studied in-vivo.

## 3. Cardiac Differentiation

Cardiovascular diseases (CVDs) are the greatest cause of mortality among non-communicable and communicable diseases [50]. As such, modelling CVDs in vitro is of great importance to better understand these diseases and to develop new drugs and alternative therapies.

Human CMs can be isolated from patient-derived heart tissue specimens, but the possibility to have access to human cardiac biopsies is rare. Moreover, current protocols to obtain adult primary CMs are still technically challenging making it difficult to obtain large quantities of viable cells. Additionally, after 24–48 h of being kept in culture, in the absence of mechanical and electrical stimuli and of supporting cells (i.e., cardiac fibroblasts), CMs undergo de-differentiation, lose their sarcomeric structure and die [51,52].

The possibility to derive hiPSC-CMs, starting from minimally invasive bioptic samples such as skin tissue, enables the creation of an in-vitro disease- and patient-specific model suitable for preclinical drug screening [53,54], thus replacing non-human cellular and animal models. Indeed, there are several challenges with these models, including their poor predictive capacity owing to inter-species differences in cardiac electrophysiology and human biology [55]. In addition, cell lines such as CHO and HEK293 cells are not ideal models for cardiotoxicity because ectopic expression of a cardiac ion channels does not always recapitulate the physiology of human CMs [56,57].

The initial observation that stem cells could mature into beating CMs was reported when ESCs were first cultured in suspension. These cells spontaneously formed three-dimensional aggregates and inside these “embryoid bodies” (EBs) cells with functional and electrical properties of CMs could be found [58]. A similar process occurring with iPSCs was later reported [59]. Even if it is rather inefficient (~1% purity of CMs) and highly cell line-dependent, the EB method is currently being applied because of its simplicity.

Another method for cardiac differentiation was inspired by embryological cardiovascular development, where the anterior endoderm has a central role in the induction of cardiac mesoderm [60,61,62]. This method is based on the coculture of iPSCs with END-2 cells, an endoderm-like cell line from mouse carcinoma cells, which may result in the formation of beating clusters [61,63,64,65,66,67,68,69,70]. The preparations resulting from this protocol have a 20–25% purity of CMs.

Different signaling pathways and growth factors have been found involved in successfully inducing cardiac mesoderm in culture [71,72,73]. Combinations of *BMP4*, *Wnt3a*, and *Activin A* induce gastrulation-like events in iPSCs cultured in a high-density monolayer with a serum- and feeder cell-free system [74]. Spontaneously contracting areas are generally observed after 10 days from induction with BMP/Activin A and, after three weeks, these cell preparations typically consist of ~30% CMs [75]. A similar protocol uses factors that activate the canonical Wnt/β-catenin signaling pathway instead of BMP/Activin *A* to induce cardiac mesoderm [76,77,78]; this methodology has been described to produce up to 50% CMs [79]. Since all these growth factors don’t elicit optimal transcript levels to induce cardiogenesis if used outside the right time frames [80], time-dependent media supplementation is crucial to obtain an efficient lineage-specific differentiation. Commercial kits provide standardized and simplified protocols to increase the reproducibility of the differentiation process [54,81].

## 4. Functional Properties of hiPSC-CMs: Overview and Limitations

The spontaneous beating that appears at the beginning of the differentiation process is generally accepted as sign for the expression, within newly developing hiPSC-CMs, of functional cardiac ion channels and transporters related to generation of action potential (AP) and contractility. Unfortunately, hiPSC-CMs generated with current protocols are still quite immature and existing differentiation techniques appear to work efficiently only with specific cell lines [82,83,84].

The characterization of electrophysiological properties of differentiating, beating CMs is key to define the level of electrical and mechanical cell maturation. Several ionic currents have been characterized in single hiPSC-CMs by using the patch-clamp technique, such as the sodium (I_Na_), the calcium (I_Ca,L_ and I_Ca,T_) and the potassium ones (I_to_, I_Kr_ and I_Ks_) [85,86,87,88,89,90]. In particular, sodium and calcium inward components contribute to the depolarizing phases of the electrical activity; while the former is responsible of the fast depolarizing process, the latter has a functional role during the slower depolarization of spontaneous automatic cells together with I_f_ pacemaker current, or during the plateau in stimulated AP, critical phase for the cell contraction. Otherwise, repolarizing process is due to the outward potassium current contribution of the AP. The balance between inward and outward currents determine the AP duration (APD) and then the refractoriness period, that are crucial in developing arrhythmic events.

The biophysical properties that characterize voltage dependence and activation/inactivation kinetics of each of these ion channels have been studied in relation to time of culture. Furthermore, their current density was found to increase from day 30 to 80 of the differentiation process. Consequently, temporal changes of these properties determine different ionic contribution to the cardiac AP (I_Na_, I_CaL_, I_K1_), leading to heterogeneous AP profiles and parameters (diastolic membrane potential, E_diast_; AP amplitude, APA; AP duration, APD) [91,92,93].

Based on the AP properties, CMs deriving from a single clone of differentiating iPSCs, frequently results in a mix of cells that can be classified as atrial-, ventricular- and nodal-like CMs [53,59,86]. However, this kind of classification is biased by being operator-dependent and may result in misleading interpretation when comparing CMs with prolonged APD (e.g., hiPSC-CMs from Long QT Syndrome patients) to healthy ones. In this context, tools can be used to identify and/or isolate atrial- or ventricular-like hiPSC-CMs. Recently, Schwach et al. have described a specific marker which is highly enriched in human atrial CMs, but not in ventricular ones, the so called chick ovalbumin upstream promoter transcription factors I and II (COUP-TFI and II) that regulates atrial-specific ion channels gene expression such as KCNA5 encoding K_v_ 1.5 (I_Kur_ current) and KCNJ3 encoding K_ir_ 3.1 (I_KACh_ current) [94,95,96]. By fusing this promoter with fluorescent reporter genes (mCherry) and combining it with the well-established human cardiac NKX2.5EGFP/+ reporter, they were able to sort a pure atrial cell population [97].

**Figure 2:**
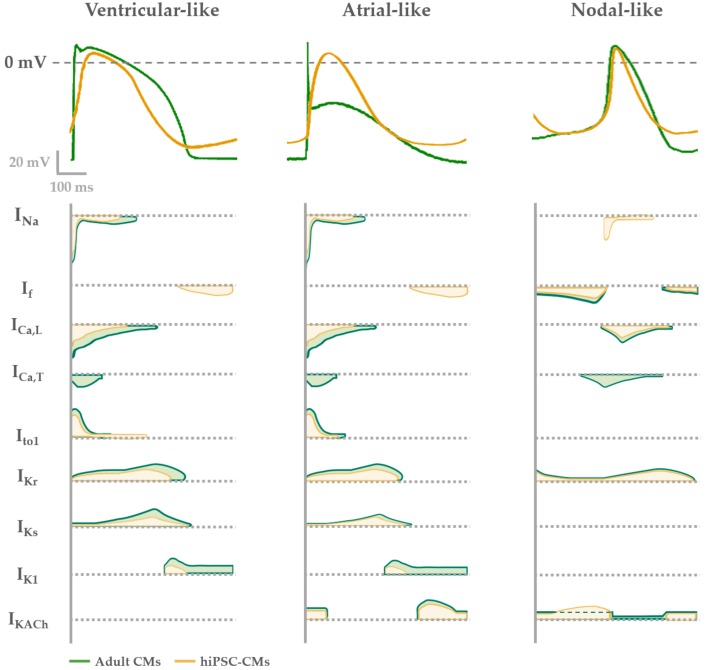
Electrophysiological phenotypes of hiPSC-derived (yellow) compared with adult CMs (green). AP shape (upper panel) described in each phenotype (ventricular-, atrial- or nodal-like) is determined by different contribution of cardiac ion currents, represented over time in the lower panel.

In Figure 2 the typical features of adult human CM APs are compared to the ones of hiPSC-CMs. In general, nodal-like hiPSC-CMs and sinoatrial CMs APs are comparable, showing spontaneous electrical activity thanks to the contribution of the funny (I_f_) and calcium (I_CaL_) currents and the absence of the inward-rectifier potassium channels (I_K1_) that usually maintains negative E_diast_. Major differences between adult and hiPSC-CM AP shapes are present when atrial and ventricular APs are analyzed. Indeed, hiPSC-CMs show more depolarized E_diast_ and they often still have a spontaneous electrical activity, because I_f_ is still functional and the I_K1_ expression is not enough to maintain an hyperpolarized E_diast_ [98]. As a consequence of the depolarized E_diast_, AP upstroke velocity and APD in hiPSC-CMs are not superimposable to those of adult CMs.

To overcome the lack of I_K1_ expression, the overexpression of Kir 2.1, I_K1_ encoding gene, [99] or an “electronic” maturation by injection of computational I_K1_ in a real time mode (dynamic clamp technique) have been designed [100,101]. In both cases, E_diast_ of derived-CMs hyperpolarizes and the activation of all expressed ion channels allows to develop an AP profile more similar to the one of atrial or ventricular adult CMs. This optimized physiological condition has been used to investigate mechanisms of cardiac cellular disease [4] and predict pharmacological approaches [5,6]. Overall, by adding I_K1_ (through dynamic clamp or channel overexpression), hiPSC-CMs AP becomes more similar to the adult one, suggesting that from the electrophysiological point of view the lack of this channel may be the main reason for the hiPSC-CM immaturity.

Additionally, hiPSC-CMs repolarization reserve is lower in comparison to adult CMs because of the low expression of the slow delayed rectifier channel I_Ks_. Indeed, the functional contribution of this current to the hiPSC-CM AP has been usually seen under β-adrenergic stimulation and reduced repolarization reserve by blocking the rapid component I_Kr_ [102,103,104]. Only in few papers I_Ks_ has been recorded in basal conditions in hiPSC-CMs [4], a sign of a good cell maturation level. For this reason, the expression of I_Ks_ together with the one of I_K1_ in hiPSC-CMs are usually seen as functional maturation markers of these cells.

**Figure 3:**
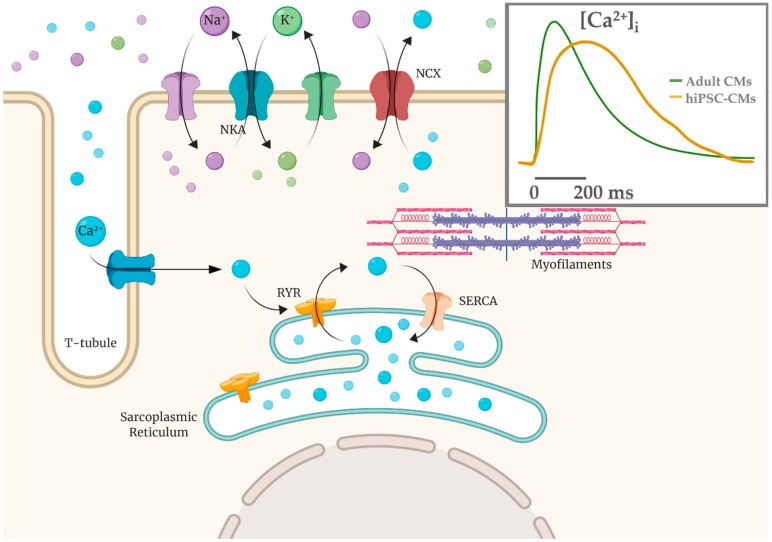
Calcium-induced calcium release mechanism (CICR) schematized with T-tubule and sarcomere structures. Ca^2+^ influx via the L-type calcium channels is able to cause a release of the SR Ca^2+^ store via the Ca^2+^-sensitive ryanodine receptors (RYR2). In hiPSC-CMs the Ca^2+^ entry is mainly the extracellular one and calcium handling kinetics are slower (yellow in the inset) compared to adult CMs (green).

Several works have studied Ca^2+^ handling proteins (L-type Ca^2+^ channels, RyR2 in sarcoplasmic reticulum, SERCA2a pump-based Ca^2+^ uptake) and Ca^2+^ transient parameters, as well SR Ca^2+^ release events (Ca^2+^ sparks) [19,105,106]. In hiPSC-CMs there is an immature condition due to a poorly developed sub-cellular T-tubules system and sarcomeric structure; these are crucial elements for Ca^2+^-handling, contractile force and relaxation processes [10,107,108,109,110]. U-shaped Ca^2+^ transients in hiPSC-CMs suggest the presence of an immature functional excitation-contraction (EC) coupling compared to native CMs (Figure 3), thus implying that kinetic properties of calcium handling process are slower compared to the adult CMs [106,111].

Single cells recordings with the patch clamp technique are still the most informative and accurate technique to disclose mechanisms underlying abnormal electrical activity in hiPSC-CMs. However, global electrophysiological information can also be acquired by the multielectrode array (MEA) system by plating spontaneous beating clusters of hiPSC-CM. This technique is useful to evaluate changes in AP rate, duration and conduction velocity.

Platforms of hiPSC-CM to test drug safety by analyzing their proarrhythmic effects have been recently developed. Cardiac electrophysiology models have been applied more and more in the emerging discipline of quantitative system pharmacology (QSP) for cardiac safety prediction [112]. hiPSC-CMs have been applied in screening the proarrhythmic potential of drugs; Sotalol, Dofetilide, and E4031 for hERG channel blockade, Quinidine and Flecainide as sodium channel blockers, and Verapamil and Diltiazem as calcium channel inhibitors represent the main examples [113,114,115]. These drugs, in conjunction with in silico modelling, have been indeed the major focus of the FDA’s Comprehensive In Vitro Proarrythmia Assay (CiPA) initiative [116]. In the last years, the CiPA has been a remarkable initiative that uses in silico models for the assessment of potential proarrhythmic effects of drugs that are then classified into high, intermediate and low risk for Torsade de Pointes (TdP) tachycardia. In particular, the potential torsadogenic effect of drugs is based on hERG (I_Kr_) or I_Ks_ (slow-delayed rectifier) outward currents inhibition with or without Nav1.5 (transient/late sodium currents, I_Na_/I_NaL_) and Cav1.2 (L-Type Ca^2+^) inward currents enhancement. The result is a delayed AP repolarization with increased incidence of early afterdepolarization (EADs), leading to ventricular arrhythmias such as TdP and ventricular fibrillation [112,117,118,119]. Furthermore, a computational approach has been recently developed to recapitulate the human AP profile and drug-induced TdP [120,121,122].

The CiPA in silico system represents a predictive strategy applied on hiPSC-CMs for development of therapeutic drugs potentially safety in term of cardiac function. Anyway, care must be taken with conclusions about healthy and pathological phenotypes of CMs, that may result misleading because of their immature functional state [89,92,123].

## 5. Pluripotency and Cardiac Differentiation of hiPSCs Derived from Cardiac vs. Non-Cardiac Sources

The reprogramming process can be applied to all type of somatic cells, such as placenta [26] mononuclear cells from blood [25], and keratinocytes [28], from which it is possible to address the differentiation process toward cardiac phenotype. The somatic source may influence the phenotype of iPSCs by affecting both reprogramming and differentiation efficiency. For example, it has been shown how blood-derived iPSCs differentiate into hematopoietic cells more easily in comparison to fibroblast-derived ones [7]; in addition, beta cell derived-iPSCs were more prone to differentiate into insulin-producing cells if compared to ESCs [124].

**Figure 4:**
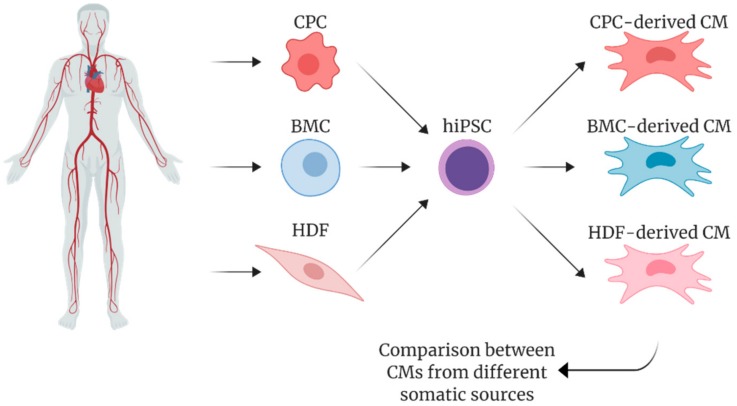
Schematic overview of human induced pluripotent stem cells (hiPSCs-CMs) generated from different somatic sources: cardiac progenitor cells (CPCs) from cardiac tissue, bone marrow cells (BMs) from sternal region, and dermal fibroblasts (HDFs) from skin.

In agreement with these observations, it has been recently reported the possibility to reprogram explant-derived cells, elsewhere referred as cardiac progenitor cells (CPCs) [125], from human cardiac biopsies obtain functional and terminal differentiated CMs [29]. As schematized in Figure 4, CPC-derived hiPSCs account for improvements in differentiation to CMs in comparison to patient-matched hiPSCs from other somatic sources, such as bone marrow-derived mesenchymal stem cells (BMC) and dermal fibroblasts (HDF) both if cultured in monolayers [126] or EBs [127,128].

These works emphasized the existence of an epigenetic memory retained by iPSCs from their tissue of origin. Reprogramming of somatic cells to pluripotency undergo a reversal in DNA modifications that characterize the cell development, but in some cases these modifications remain unaltered, representing a residual tissue-specific DNA methylation that influences the differentiation potential of iPSCs [129].

Although hiPSC derived from HDFs have been described to produce a higher number of colonies that appear earlier in time, the expression level of pluripotency markers (e.g., *Nanog*, *Oct4*) resulted significantly enhanced in hiPSC from CPCs as compared to both hiPSC from HDFs and from BMCs [126,128]. Inversely, Sanchez-Freire et al. [127] showed that the expression of pluripotency markers was not different between the two hiPSC lineages from different tissues. In both cases, the ability of reprogrammed cells to form three germ layers (i.e., mesoderm, ectoderm, and endoderm), which is considered a hallmark for pluripotency in iPSCs, is not affected by the cell source.

As for the specification potential toward cardiac phenotype, hiPSC derived from cardiac somatic sources showed higher efficiency during the re-differentiation process compared to non-cardiac ones in terms of genes expression for early (NKX 2.5, ISL1) and late cardiogenic transcription factors (HAND2, TBX5, GATA4 and MEF2C) [127,128]. Genes encoding for late cardiac specific markers, such as MYLC2.a, MYH6, TNNI3 and TNNT2, were also overexpressed in cardiac hiPSC-CMs, as well as those encoding for cardiac specific ion channels (HCN1-4, CACNA1C and 1G, RyR2, Cx43) [126,128].

Accordingly, a higher percentage of Troponin T (cTnT)-positive CMs in beating cardiac *Sca1*-iPSC-CMs (cardiac) compared to HDF-iPSC-CMs (non-cardiac) has been reported both by Sanchez-Freire et al. (15 days) [127] and Meraviglia et al. (18–20 days) [128] as a late differentiation marker. Taken together these data support the hypothesis that the cardiac origin of somatic cells to be reprogrammed influences the transcription of cardiac genes during the differentiation of iPSCs.

While Meraviglia et al. and Pianezzi et al. observed that hiPSC-CMs started beating at 10 days of differentiation, Sanchez-Freire et al. needed five more days to detect the first spontaneous events in their *Sca1*- and HDF-derived CMs. Furthermore, in the studies by Meraviglia et al. and Pianezzi et al. this correlated with an upregulation of cTnI expressed in a sarcomeric pattern. In addition, CPC-derived CMs from Pianezzi et al. are the first population to exhibit early spontaneous beating (at 10 days of differentiation) compared to patient-matched HDF- and BMC-derived ones (15 days), thus suggesting precocity in cell differentiation from cardiac source.

Functionally, Meraviglia et al. and Sanchez-Freire et al. did not observe any differences in term of beating rates between cardiac and non-cardiac sources derived-CMs at 30 days of differentiation. Interestingly, in urine-derived hiPSC-CMs [111] the adaptation of AP to stimulation rates was not observed until 90 days of maturation, while in our hand CPC-derived cells showed APD_90_ shortening when stimulated from 2 to 4 Hz already at 35 days of differentiation (unpublished). Meraviglia et al. noticed that the maturation process affected especially the maximum diastolic potential (MDP) values, that resulted more hyperpolarized in CPC-CMs at 60 day of differentiation. However, Sanchez-Freire et al. did not observe any electrical difference between cardiac- and fibroblast-derived CMs at day 30.

In recent work, it has been observed variability in electrical properties and sensitivity to ion channel blockers in CMs derived from different sources [130]. Accordingly, the MEA measurements by Pianezzi et al. pointed out a higher maturation degree of CPC-CMs by highlighting the presence of I_Ks_, a current more expressed and more functional in CPC-derived CMs in comparison to HDF- and BMC-derived cells. A higher repolarization reserve in CPC-CMs has been demonstrated by highlighting the contribution of I_Ks_ with the specific blocker JNJ303 under I_Kr_ blockade with E4031. In support of this, a JNJ303-dependent QT prolongation resulted strongly enhanced in CPC-CMs in comparison to HDF- and BMC-CMs [126].

In Pianezzi et al. CMs derived from cardiac somatic cells showed differences from an early stage of maturation in calcium handling. Here, not only CPC-, but also HDF- and BMC-CMs at 35 days of differentiation were able to elicit RyR-mediated Ca^2+^ release when exposed to caffeine. However, the quantification of the number of responsive CMs clearly showed that the percentage of CPC-derived ones was significantly greater than the percentages of CMs derived from the other two cell types. On the other hand, the molecular expression of RyR2 and SERCA2a proteins were not different among the three groups. Thus, the “caffeine responsiveness” may represent a functional index, over the expression of cardiac specific genes, for the identification of differentiating CMs. Despite of this, in Sanchez-Freire et al., 30 days of differentiation were not sufficient to evince any differences between CPC- and HDF-CMs, equally immature in Ca^2+^ transient properties.

In general, we can say that, although different somatic cells show a cardiogenic potential when exposed to appropriate cardiac stimuli, cardiac precursor cells seem to be temporally and/or qualitatively more prone to differentiate into functional cardiac cells. Moreover, it must be clarified whether maturation of reprogrammed cells from cardiac sources represents also at late time points a better cellular substrate for cardiac disease modelling, drug testing and tissue regeneration.

## 6. Conclusions

To date, it has been widely described how hiPSC-CMs are able to recapitulate molecular and functional aspects of human heart pathophysiology, thus providing a good tool for disease modelling and development of personalized therapy that involves a pharmacological treatment. A wide range of genetic cardiomyopathies has been modelled using hiPSC-CMs [131], for example familiar long QT (LQT) syndromes [4,85,86,87,132,133], Brugada syndrome [134,135], Catecholaminergic polymorphic tachycardia (CPVT) [136,137] and atrial fibrillation [138,139].

Unfortunately, the physiological phenotype of iPSC-CMs is heterogeneous both in term of sub-populations of CMs and in term of maturation degree during differentiation protocol, potentially leading to an incorrect interpretation of data. To avoid this, the comparison of their functional parameters with the native and adult counterpart is crucial. The cellular size and morphology, together with the expression of structural proteins and a T-tubular system that ensure the electrical conduction, must be evaluated in order to perform accurate functional analysis and develop 3D platforms; electrophysiological parameters and Ca^2+^ handling features, contractile force, responses to beta-adrenergic stimulation, metabolic profile and conduction velocity must be verified to assess the ability of hiPSC-CM-based models to recapitulate diseases and pathological phenotypes. Furthermore, populations of cells differentiated from iPSCs contain non-cardiomyocyte cells that may interfere with maturation levels, electrophysiological properties and conduction velocity of differentiating CMs, therefore affecting the sensitivity to tested drugs. Standardization of methods and techniques from one laboratory to another is needed for a reliable comparison between healthy and pathological cell models.

Current differentiation protocols that are being tested to optimize the structural and functional maturation degree of hiPSC-CMs use addition of physiological substrates, prolongation of culture time, coculture with endothelial cells or fibroblasts, 3D cell platforms (“organoids”) and mechanical and electrical stimulation (dynamic clamp); these techniques, combined with purification methods such as pre-plating or substitution of glucose with lactate in the early maturation phase of CMs, can produce up to 90% cTnT-positive hiPSC-CMs [140,141,142,143,144,145,146,147].

Despite their limitations, thanks to molecular, structural and functional correlations with primary adult CMs, hiPSC-CMs can be considered reliable tool for disease modelling and it represents a valid platform for pharmacological screening [53,54]. Moreover, it is crucial to consider the somatic origin of hiPSC-CMs since it has been clearly demonstrated to impact on time of development and maturation degree of derived CMs in a patient-matched comparison.

The selection of a somatic donor tissue has to be adjusted according to the goal of the study. Since CPCs are derived from cardiac biopsies of patients who undergo heart surgery, the accessibility to human material can be limited. For these reasons, the use of cardiac derived cells as source to generate hiPSCs represents a compromise between the possibility to obtain a more mature CM and the invasiveness and risks of cardiac procedures.

## Abbreviations

hiPSC	Human Induced Pluripotent Stem Cell
CM	Cardiomyocyte
ESC	Embryonic Stem Cell
MET	Mesenchymal to Epithelial Transition
CVD	Cardiovascular Disease
CPC	Cardiac Progenitor Cell
BMC	Bone Marrow-derived stem Cell
HDF	Human Dermal Fibroblast
Na^+^	Sodium
K^+^	Potassium
Ca^2+^	Calcium
E_diast_	Diastolic Membrane Potential
AP	Action Potential
APA	Action Potential Amplitude
APD	Action Potential Duration
MDP	Maximum Diastolic Potential
MEA	Multielectrode Array
ECG	Electrocardiogram
TdP	Torsade de Pointes
LQTS	Long QT Syndrome
CPVT	Catecholaminergic Polymorphic Ventricular Tachycardia
DCM	Dilated Cardiomyopathies
HCM	Hypertrophic Cardiomyopathies
QSP	Quantitative System Pharmacology
CiPA	Comprehensive In Vitro Proarrythmia Assay
